# Cyclophosphamide-induced testicular injury: the role of chrysin in mitigating iron overload and ferroptosis

**DOI:** 10.1007/s00210-024-03519-4

**Published:** 2024-11-20

**Authors:** Dalia O. Saleh, Nesma M. E. Abo El Nasr, Yosra A. Hussien, Marawan Abd El-Baset, Kawkab A. Ahmed

**Affiliations:** 1 Pharmacology Depatrment, Medical Research and Clinical Studies Institute, National Research Centre, Giza, Egypt; 2 Pathology Department, Faculty of Veterinary Medicine, Cairo University, Giza, Egypt

**Keywords:** Chrysin, Cyclophosphamide, Testicular toxicity, Iron overload, Ferroptosis

## Abstract

This study evaluated the beneficial effects of chrysin against cyclophosphamide (CP)-induced testicular toxicity in rats across several parameters, including hormones, oxidative stress, inflammation, apoptosis, and protein expression. Rats were pretreated with oral doses of chrysin at 25, 50, or 100 mg/kg daily for 7 days. On the 8th day, all groups except controls received CP (200 mg/kg) injection. Chrysin doses continued for 7 more days. Hormones, oxidative stress markers, inflammatory cytokines, apoptosis regulators, and iron regulatory proteins were assessed. CP decreased testosterone, inhibin B, GSH, and GPx4 and increased FSH, cholesterol, MDA, IL-6, and BAX. It also drastically reduced TfR1, liprin, and IREB2. Chrysin dose-dependently counteracted these effects. The highest 100 mg/kg chrysin dose increased testosterone, inhibin B, GSH, GPx4, BCL2, TfR1, liprin, and IREB2 while decreasing FSH, cholesterol, MDA, IL-6, and BAX close to control levels. There were also significant incremental benefits for testosterone, inhibin B, and other parameters with higher chrysin doses. Chrysin dose-dependently attenuated CP-induced hormonal dysfunction, oxidative stress, inflammation, apoptosis, and iron-regulatory protein suppression. The maximum dose showed the most optimal protective effects in restoring the testicular toxicity markers. These results validate the promising spermatoprotective properties of chrysin against chemotherapeutic germ cell damage.

## Introduction

Cyclophosphamide (CP) (N, N-bis (2chloroethyl)-2-oxo-1,3,2λ5oxazaphosphinan-2-amine) as a bifunctional alkylating cytotoxic agent is one of the most invasive chemotherapeutic agents, which commonly used as an antineoplastic drug to treat various malignant tumors and as an immunosuppressive agent (Parandin et al. 2023). Unfortunately, it is non-selective for cancer cells, and it may lead to toxic adverse events in numerous organ systems involving the testicular dysfunction causing male infertility in both animals and human (Hosseini et al. 2018; Khamis et al. 2023).

CP is transformed into its active metabolites, namely, phosphoramide mustard and acrolein, by the hepatic microsomal cytochrome P450 mixed function oxidase system, while phosphoramide mustard is related to the antineoplastic and immunosuppressive actions of CP alongside acrolein is associated with harmful side effects including apoptosis as well as necrosis in normal tissue (Watcho et al. 2019; Rezaei et al. 2021). Acrolein impairs metabolic processes and induces oxidative stress in tissues, which lowers fertility in patients receiving therapy (Ayegbusi et al. 2023). In fact, acrolein interacts with DNA to cause aberrant cellular activity, necrosis, and cell death. Furthermore, it has been found in recent research that acrolein causes male infertility by increasing spermatogenic cell death and inhibiting testosterone production (Watcho et al. 2019). CP has been reported to affect male fertility both centrally and peripherally by downregulating the hypothalamic–pituitary–gonadal (HPG) axis. It does this by impacting hypothalamic Kiss1 mRNA expression and gonadotropin secretion, disrupting testicular steroidogenesis and testosterone synthesis, and ultimately leading to azoospermia and impaired spermatogenesis (Rezaei et al. 2021; Khamis et al. 2023).

The gonadotoxicity of this alkylating cytotoxic chemical causes male infertility by interfering with the testicles’ ability to produce sperm. In addition, studies conducted on male rats and mice given CP have shown that CP treatment results in decreased testicular weight, temporary oligospermia, abnormal changes to sperm motility and fertilization capacity, and abnormal changes to the testis and epididymis (Busı et al. 2023). Furthermore, CP has been associated with direct testicular oxidative stress and DNA damage, resulting in testicular degeneration (Ebokaiwe et al. 2022; van den Boogaard et al. 2022), hence a variety of detrimental consequences of CP on reproductive function in both humans and experimental animals.

Ferroptosis, a type of programmed iron-dependent non-apoptotic cell death, impacts testicular function by promoting lipid peroxidation, and inflammation, leading to cell death in key testicular cell populations (Su et al. 2022). This cell death disrupts spermatogenesis and steroidogenesis, resulting in impaired sperm production and reduced testosterone levels. Understanding the relationship between ferroptosis and testicular function highlights the importance of maintaining iron homeostasis and antioxidant defenses to protect against testicular dysfunction (Yang et al. 2022). Iron dysregulation is a key trigger for ferroptosis, driven by iron-dependent lipid peroxidation. Insufficient iron uptake can lead to cellular iron overload in specific compartments, contributing to oxidative damage and ferroptotic cell death within the testes (Feng et al. 2023).

Ferroptosis involves excessive intracellular ferrous accumulation, resulting in the depletion of glutathione (GSH), the inactivation of glutathione peroxidase 4 (GPX4), and the upregulation of lipid peroxidation. Iron is vital for spermatogenesis and male reproductive function, but there is insufficient data to fully support the involvement of ferroptosis in testicular toxicity. The precise molecular mechanisms by which ferroptosis induces damage in the testes remain unidentified (Ling et al. 2023; Yuan et al. 2023).

Chrysin is a natural flavone found in various dietary sources with numerous medicinal and pharmacological properties. It is abundant in several plant species, fruits, and mushrooms, including passion flowers, Propolis, honey, carrots, chamomile, and mushrooms (Pleurotus ostreatus) (Ye et al. 2022). Various studies were underlined the pharmacological potential of Chrysin as an antioxidant, anticancer, anti-inflammatory, hepatoprotective, cardioprotective, neuroprotective, and nephroprotective agent as well as uro-protective effect against hemorrhagic cystitis induced by CP (Ye et al. 2022; Saleh et al. 2023).

The aim of this study is to explore the potential therapeutic effects of chrysin against the progression of CP-induced testicular toxicity in rats, with a specific focus on targeting ferroptosis. This investigation aims to provide a promising strategy for preserving reproductive dysfunction associated with CP medication. Specifically, the study evaluates the impact of chrysin on serum parameters, including testosterone, inhibin B, and follicle-stimulating hormone (FSH) levels, as well as testicular cholesterol. Testicular inflammatory markers such as interleukin-6 (IL-6), BAX, and BCL2 were also assessed. Additionally, the study examines oxidative stress markers including malondialdehyde (MDA), glutathione (GSH), and glutathione peroxidase 4 (GPx4) and assesses the effect of chrysin on protein expression levels of iron-responsive element-binding protein 2 (IREB2), transferrin receptor 1 (TfR1), and liprin in the testes of CP-treated rats. Furthermore, the study investigates sperm count and quality parameters, including relative testis weight, live and dead sperm percentages, fructose content, and sperm motility. By evaluating these parameters and investigating the various molecular signaling cascades involved in protecting male fertility, the study aims to elucidate the potential ameliorative role of chrysin in mitigating the adverse effects of CP-induced testicular damage.

## Materials and methods

### Animals

The study protocol adhered to the ethical guidelines set forth by the National Research Centre’s (NRC) Ethical Committee in Cairo, Egypt (Approval No. 01480324), and followed the Guidelines for Ethical Conduct in the Care and Use of Nonhuman Animals in Research of the American Psychological Association. Adult male Wistar rats were procured from the NRC animal house in Cairo, Egypt. Rats underwent a 1-week acclimatization period and were housed under standard conditions (60% relative humidity with a tolerance of ±10%, temperature maintained at 23 °C with a tolerance of ±2 °C, and a 12-h light/dark cycle), with ad libitum access to food and water.

### Chemicals and drugs

CP and chrysin were obtained from Sigma-Aldrich Company in St. Louis, Missouri. Additionally, only molecular or analytical-grade compounds were used.

### Experimental design

The study utilized 36 male Wistar rats weighing between 138 and 155 g. The experimental groups were structured as follows (*n* = 6):

- Group 1 (control): Received intraperitoneal (I.P) administration of saline (0.2 ml) on the 8th day of the experiment.
- Group 2 (CP): Saline was injected orally by oral gavage for seven consecutive days. On the 8th day, a single intraperitoneal (IP) dose of CP at 200 mg/kg was administered. Following this, saline was again administered orally for another 7 days.
- Group 3 (CP+ chrysin25): Chrysin was orally administered by oral gavage at a dosage of 25 mg/kg for 7 consecutive days. On the 8th day, a single intraperitoneal dose of CP at 200 mg/kg was administered. Then, chrysin was orally administered again at 25 mg/kg for another 7 days.
- Group 4 (CP+ chrysin50): Chrysin was orally administered at a dosage of 50 mg/kg for 7 consecutive days. On the 8th day, a single intraperitoneal dose of CP at 200 mg/kg was administered. Then, chrysin was orally administered again at 50 mg/kg for another 7 days.
- Group 5 (CP chrysin100): Chrysin was orally administered at a dosage of 100 mg/kg for 7 consecutive days. On the 8th day, a single intraperitoneal dose of CP at 200 mg/kg was administered. Then, chrysin was orally administered again at 100 mg/kg for another 7 days (Saleh et al. 2023).

### Blood and tissue sampling

Rat blood samples were withdrawn from the tail vein and then centrifuged at 3000 revolutions per minute (rpm) for a duration of 15 min. The serum was collected and kept at – 20 °C for hormonal and biochemical assessment of Testosterone (Fine test, Catalogue No.: ER1462, China), inhibin b (Elabscience, Catalogue No: E-EL-R1027, USA), FSH (Abnova, Catalogue No: KA2330, Taiwan), and cholesterol (Bio vision, Catalogue No: K4436-100, USA). After that, the rats were sacrificed by cervical dislocation. Testes were isolated and washed in saline solution. The right testes were kept in 10% formalin for further histological investigation, while the left testes were frozen at – 80 °C to assess the following parameters using the homogenate/malondialdehyde (MDA) level (Bio vision, Catalogue No: K739-100, USA), glutathione (GSH) level (Bio vision, Catalogue No: K464-100, USA), interleukin-6 (IL-6) (Cloud Clone, Catalogue No SEA079Ra, USA), GPX4 (BT lab, Catalogue No: E1787Ra, China), BAX, and BCL2 and expressed in term of mg protein. Transferrin receptor 1(TfR1), LAR protein-tyrosine phosphatase-interacting protein (liprin) and iron response element binding protein 2 (IREB2) (Catalog Number:23829-1-AP, USA) were estimated by western blot relative to beta-actin.

### Histopathological examination

Testes were obtained, dissected soon after sacrifice, and used for histological examination. After scarification of rats, the testes were removed and used for histological analysis. Harvested testes of rats from all experimental groups were fixed in neutral buffered formalin 10%, then subjected to dehydration using ascending grades of ethyl alcohol, cleared in xylene, and embedded in molted paraffin wax. Then, 4–5-μm-thick sections were prepared and stained with Hematoxylin and Eosin (H & E) (Bancroft et al. 2004) for histopathological examination. The stained sections were inspected blindly by pathologist under a light microscope (BX43, Olympus) and photographed using the Cellsens dimension software (Olympus) connected to the Olympus DP27 camera. The testicular scoring system was done according to modified Johnsen’s score. This method applies a score from 10 (full spermatogenesis) to 1 (no seminiferous epithelium) for each seminiferous tubule cross-section (Farag et al. 2021). The germinal epithelium of at least 50 randomly chosen seminiferous tubules in each group under power field X200 was assessed for each testis and the mean Johnsen’s score per rat was calculated.

### Statistical analysis

Before proceeding with the statistical analysis, data values were checked for normality using the Shapiro test and for homogeneity by Brown-Forsythe test. All of the values were presented as mean ± SD. Graph Pad Prism (version 6.0; San Diego, CA, USA) was used for the statistics analysis. For multiple comparisons, the analysis of variance (ANOVA) test was used, followed by the Tukey-Kramer test as a post-ANOVA test. Findings were considered statistically significant if the *P* value was less than 0.05. Additionally, Johnson’s scoring, relative testis weight, progressive, testosterone, and fructose was assessed using the Kruskal-Wallis test, followed by Dunn’s test for non-parametric parameters and represented as median ± interquartile range.

## Results

### Effect of chrysin on sperm count and quality of CP-induced testicular injury

**Fig. 1:**
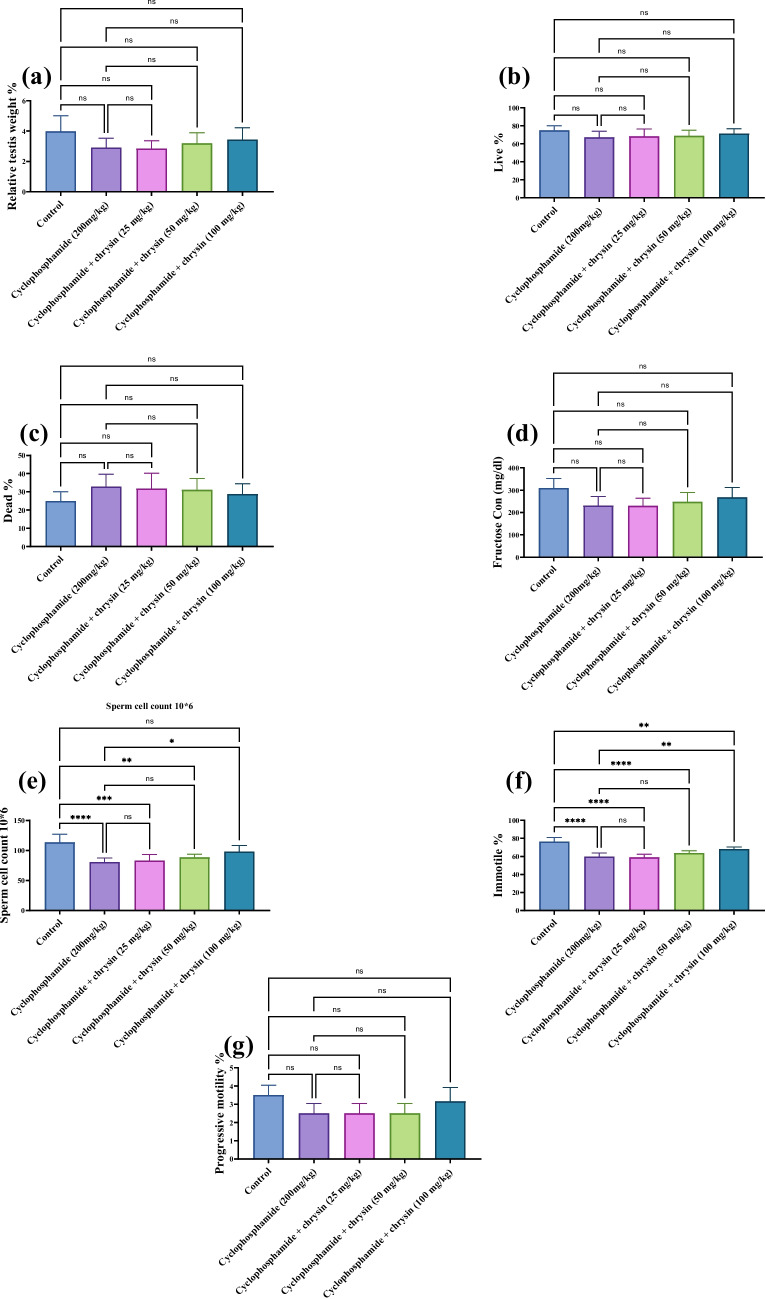
Effect of chrysin on **a** relative testis weight, **b** live percentage, **c** dead percentage, **d** fructose concentration, **e** sperm count, **f** immotile percentage, and **g** the progressive motile percentage against cyclophosphamide-induced testicular injury. Data are presented as mean ± SD. *Significant difference compared to control groups

CP treatment resulted in a 29% reduction in sperm count and a 23% decrease in non-progressive motility versus control. Chrysin dose-dependently improved these semen parameters compared to CP. The maximum restoration was seen at the 100 mg/kg dose, which increased progressive motility by 14% and the sperm count by 22% compared to the CP group (Fig. 1).

### Effect of chrysin on serum parameters testosterone, inhibin b, and FSH of CP-induced testicular injury

**Fig. 2:**
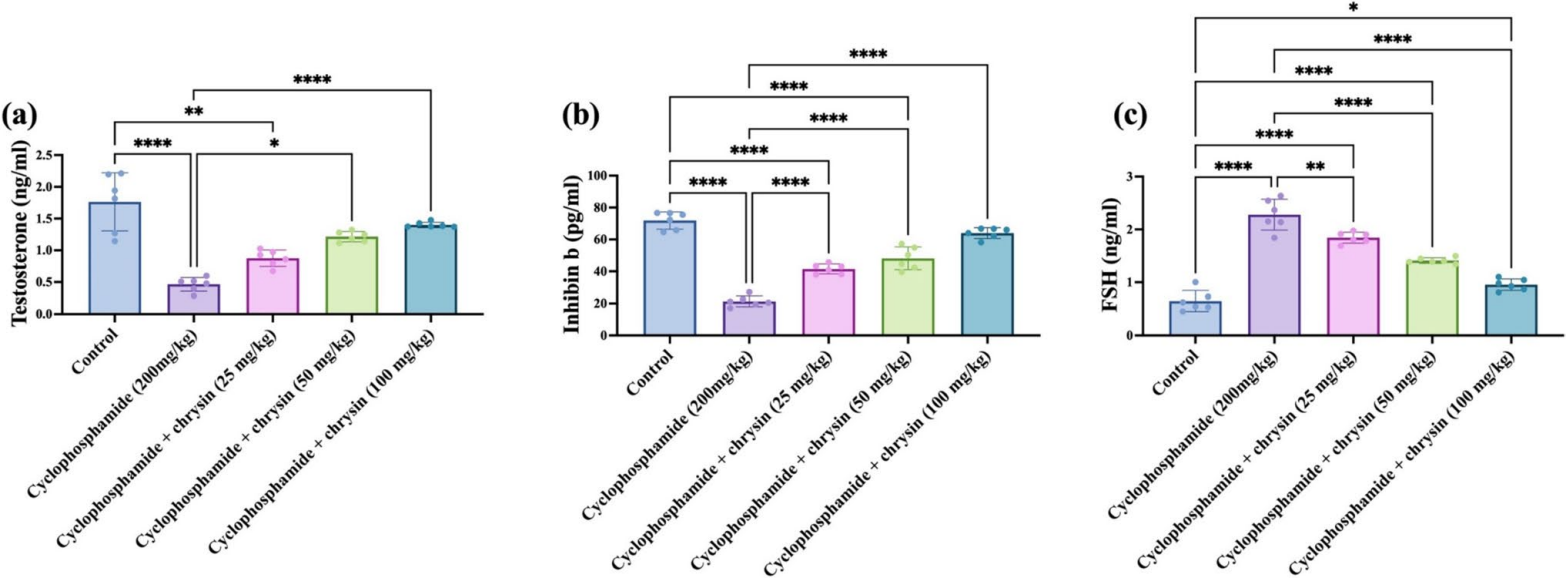
Effect of chrysin on serum parameters **a** testosterone, **b** inhibin b, and **c** FSH against cyclophosphamide-induced testicular injury. Data are presented as mean ± SD. *Significant difference compared to control groups

As shown in Fig. 2a, testosterone levels in the CP group showed a 73.86% decrease compared to control. The 50 mg/kg chrysin dose showed a greater increase of 165.22% over CP. The highest dose of 100 mg/kg chrysin increased testosterone by a very substantial 204.35% compared to CP. When comparing the different chrysin doses, the 50 mg/kg dose led to a moderate 38.64% increase over the 25 mg/kg dose. The 100 mg/kg dose showed a slight increase of 14.75% over the 50 mg/kg dose.

The CP group exhibited a 70.34% decrease in inhibin b levels relative to control. Treatment with 25 mg/kg chrysin increased inhibin b by 94.74% over CP. At 50 mg/kg chrysin, inhibin b rose even more substantially, by 126.11% compared with CP. The highest chrysin dose of 100 mg/kg led to an increase of 200.47% over CP. Comparing the different doses, the 50 mg/kg chrysin dose showed a partial increase of 16.01% versus 25 mg/kg chrysin. The 100 mg/kg dose led to a 32.87% increase compared to 50 mg/kg chrysin. Interestingly, the 100 mg/kg dose was comparable to the serum inhibin b level of the control group. The results are shown in Fig. 2b.

The CP group displayed a 2.5-fold momentous increase in FSH relative to control. Treatment with 25 mg/kg chrysin led to a significant 19.3% decrease versus CP. At 50 mg/kg chrysin, the reduction was more effective at 38.16% compared with CP. The 100 mg/kg chrysin dose showed a maximum decrease of 58.33% below CP levels. The 50 mg/kg chrysin increased the FSH level higher than 25 mg/kg chrysin. Also, there was a significant difference among the FSH serum levels of 100 mg/kg chrysin versus 50 mg/kg chrysin and 25 mg/kg chrysin. The results are displayed in Fig. 2c.

### Effect of chrysin on testicular inflammatory marker IL-6, BAX, and BCL2 of CP-induced testicular injury

**Fig. 3:**
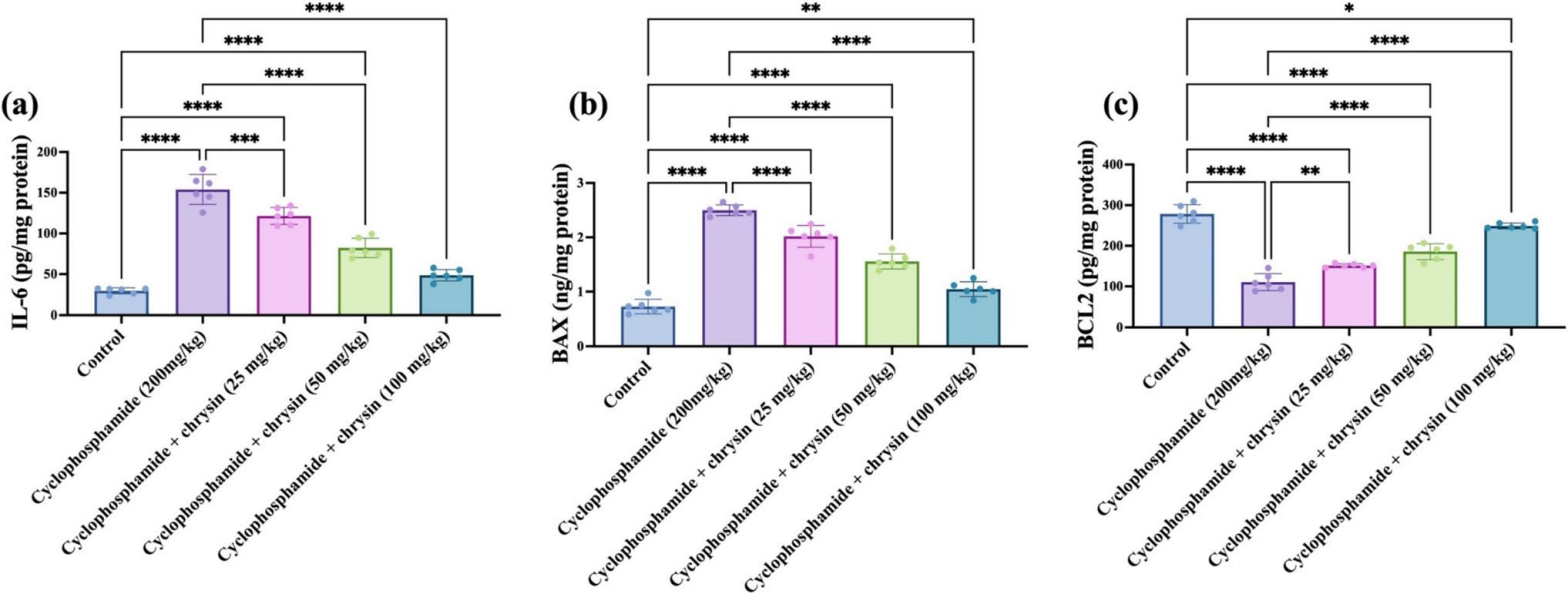
Effect of chrysin on the testicular inflammatory marker **a** IL-6, **b** BAX, and **c** BCL2 against cyclophosphamide-induced testicular injury. Data are presented as mean ± SD. *Significant difference compared to control groups

The CP group exhibited an increase of 423.6% in IL-6 relative to control. Treatment with 25 mg/kg chrysin led to a significant 21.11% reduction compared with CP. At 50 mg/kg chrysin, IL-6 declined remarkably by 46.62% below CP. The 100 mg/kg chrysin dose showed a maximum decrease of 68.37% versus CP (*p *< 0.0001). Between doses, the 50 mg/kg chrysin dose displayed a significant 32.21% lower IL-6 than 25 mg/kg chrysin (*p *< 0.0001). The 100 mg/kg dose showed an extremely substantial 40.79% decrease compared with 50 mg/kg chrysin. The results are displayed in Fig. 3a.

BAX levels with CP was elevated by 242.47% over control. Treatment with 25 mg/kg chrysin led to a significant 19.2% decrease versus CP. The reduction was more significant at 50 mg/kg chrysin at 37.6% below CP. The 100 mg/kg chrysin dose showed a maximum decrease of 58% compared to CP. Between doses, the 50 mg/kg chrysin dose displayed a significant 22.77% lower BAX than 25 mg/kg chrysin. The 100 mg/kg dose showed a significant 32.69% reduction compared to 50 mg/kg chrysin. The results are shown in Fig. 3b.

The CP group exhibited an extremely significant 60.34% decrease in BCL2 relative to control. Treatment with 25 mg/kg chrysin led to a moderately substantial 36.62% increase over CP. The increase was much more significant at 50 mg/kg chrysin at 67.94% above CP. The 100 mg/kg chrysin dose showed a maximum increase of 125.05% versus CP. Between doses, the 50 mg/kg chrysin dose displayed a small but significant 23.04% higher BCL2 than 25 mg/kg chrysin. The 100 mg/kg dose showed a massive 34.11% increase compared with 50 mg/kg chrysin as shown in Fig. 3c.

### Effect of chrysin on testicular (a) cholesterol, (b) MDA, (c) GSH, and (d) GPx4 markers of CP-induced testicular injury

**Fig. 4:**
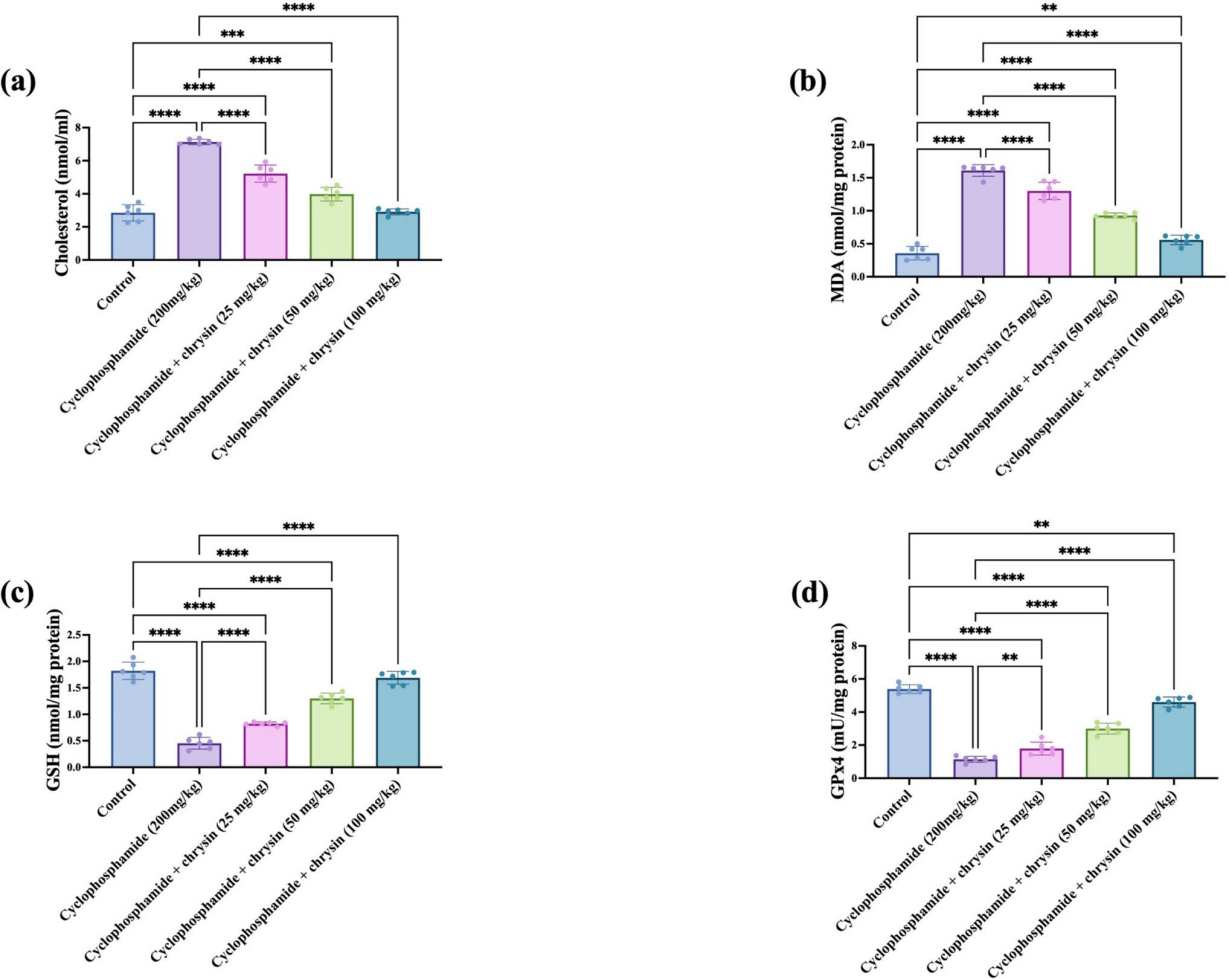
Effect of chrysin on testicular **a** cholesterol, **b** MDA, **c** GSH, and **d** GPx4 against cyclophosphamide-induced testicular injury. Data are presented as mean ± SD. *Significant difference compared to control groups

CP treatment resulted in a substantial 150.18% increase in cholesterol compared with control. The 25 mg/kg chrysin dose decreased cholesterol by 26.79% relative to CP. At 50 mg/kg chrysin, cholesterol declined by 44.18% versus CP. The 100 mg/kg chrysin dose led to a maximum decrease of 59.18% compared with CP. Between doses, the 50 mg/kg chrysin dose showed a moderate 23.76% decrease versus 25 mg/kg chrysin. The 100 mg/kg dose displayed a relatively 26.88% reduction compared with 50 mg/kg chrysin. The results are displayed in Fig. 4a.

MDA levels with CP increased by 347.22% over control. Treatment with 25 mg/kg chrysin led to a significant 19.25% decrease in MDA compared to CP. The reduction was much more prominent at 50 mg/kg chrysin at 42.24% below CP. The 100 mg/kg chrysin dose showed a maximum decrease of 65.22% versus CP. Comparing doses, the 50 mg/kg chrysin dose displayed a significant 28.46% lower MDA than 25 mg/kg chrysin. The 100 mg/kg dose had an extremely substantial 39.78% decrease relative to 50 mg/kg chrysin. The results are shown in Fig. 4b.

The CP group showed a highly significant 75.27% decrease in GSH compared with control. Treatment with 25 mg/kg chrysin remarkably increased GSH by 82.22% over CP levels. The increase was even more significant at 50 mg/kg chrysin at 1.88-fold versus CP. The 100 mg/kg chrysin dose led to the maximum increase of 2.75-fold above CP. Between doses, the 50 mg/kg chrysin dose showed a significant 58.54% higher GSH than 25 mg/kg chrysin. The 100 mg/kg dose displayed a significant 30% increase compared with 50 mg/kg chrysin as shown in Fig. 4c.

As shown in Fig. 4d, CP treatment led to a substantial 78.85% decrease in GPx4 activity relative to control. At 25 mg/kg chrysin, GPx4 activity increased by 57.02% over CP. The increase was much more significant, with 50 mg/kg chrysin at 163.16% above CP. The 100 mg/kg chrysin dose showed a maximum increase of 3.03-fold versus CP. Between the chrysin doses, the 50 mg/kg dose displayed a very significant 67.6% higher GPx4 than 25 mg/kg chrysin. The 100 mg/kg dose showed an extremely significant 53.33% increase compared to 50 mg/kg chrysin.

### Effect of chrysin on testicular Protein expression of IREB2, TFR1, and liprin of CP-induced testicular injury

**Fig. 5:**
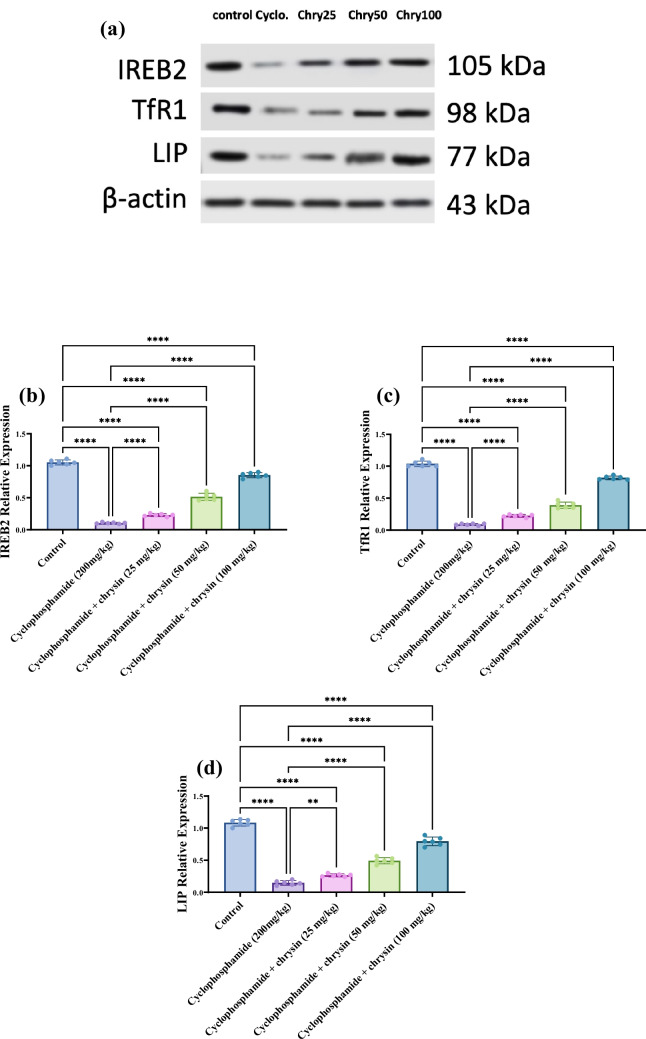
Effect of chrysin on testicular western blot **a **analyses of **b **IREB2, **c **TFR1, and **d ** Liprin against cyclophosphamide-induced testicular injury. Data are presented as mean ± SD. *Significant difference compared to control groups

CP treatment resulted in a vast 90.48% decrease in IREB2 compared with control. At 25 mg/kg chrysin, IREB2 increased substantially by 130% over CP. The increase was much more significant with 50 mg/kg chrysin at 4.10-fold above CP. The 100 mg/kg chrysin dose led to a remarkable maximum increase of 7.50-fold versus CP. Between doses, the 50 mg/kg chrysin dose showed an extremely significant 121.74% higher IREB2 than 25 mg/kg chrysin. The 100 mg/kg dose displayed a very significant 66.67% increase compared with 50 mg/kg chrysin. The results are shown in Fig. 5b. 

As shown in Fig. 5c, CP treatment resulted in a significant 91.35% decrease in TfR1 compared to control. At 25 mg/kg chrysin, TfR1 rose remarkably by 144.44% over CP levels. The increase was much more significant with 50 mg/kg chrysin at 3.33-fold above CP. The 100 mg/kg chrysin dose led to the spectacular maximum increase of 8.11-fold versus CP. Between doses, the 50 mg/kg chrysin dose showed a significant 77.27% higher TfR1 than 25 mg/kg chrysin. The 100 mg/kg dose displayed an extremely significant 110.26% increase compared to 50 mg/kg chrysin.

The CP group exhibited an 86.11% decrease in liprin relative to control. Treatment with 25 mg/kg chrysin increased liprin insignificantly over CP. The increase was even more significant at 50 mg/kg chrysin at 2.26-fold above CP. The 100 mg/kg chrysin dose led to a remarkable maximum increase of 4.26-fold versus CP. Between doses, the 50 mg/kg chrysin dose showed a significant 81.48% higher liprin than 25 mg/kg chrysin. The 100 mg/kg dose displayed a significant 61.22% increase compared with 50 mg/kg chrysin. The results are shown in Figure 5d.

### Effect of chrysin on histopathology findings of CP-induced testicular injury

**Fig. 6:**
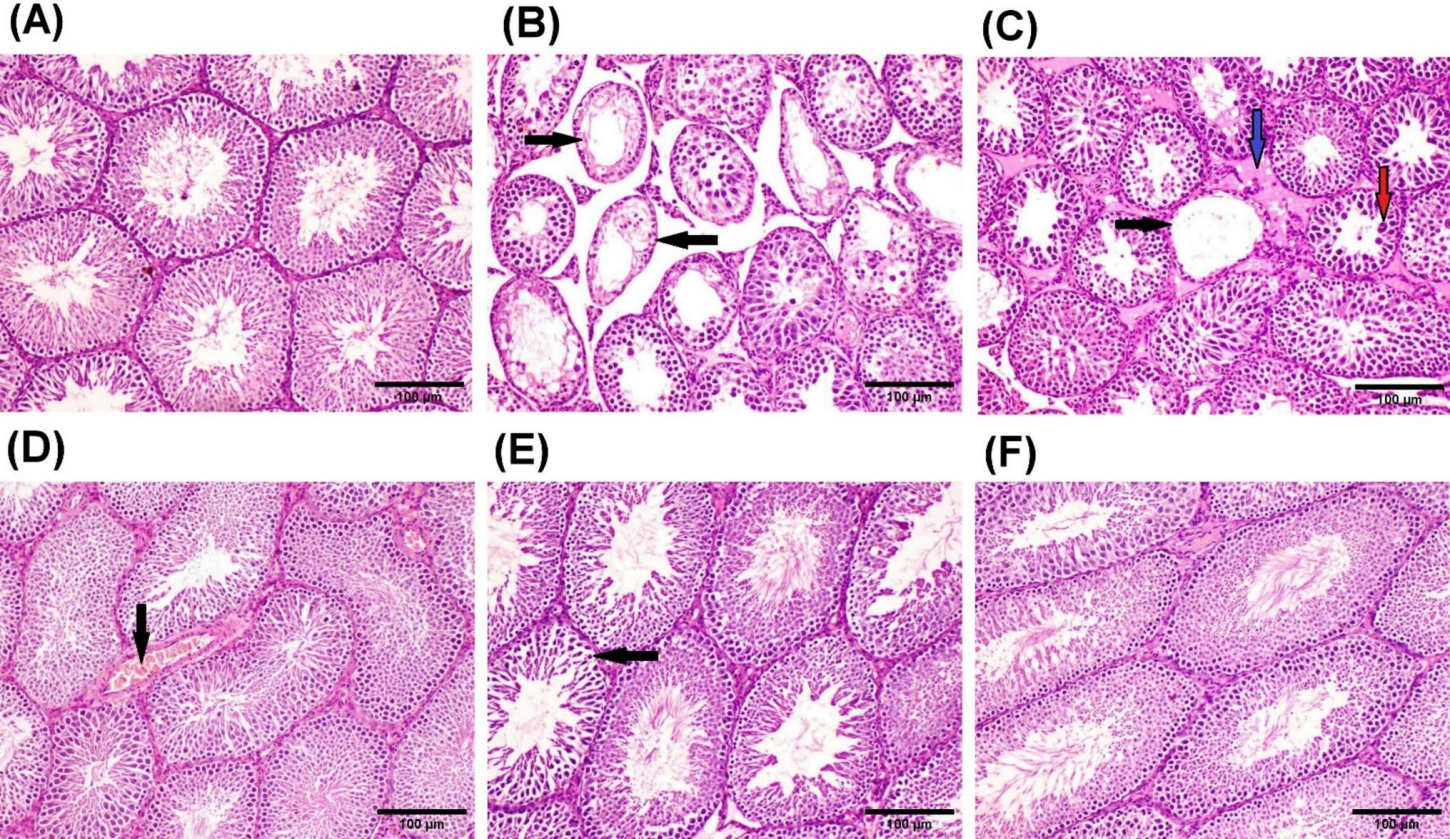
Representing photomicrographs of H & E-stained testicular sections of rats **A** control; showing normal histoarchitecture of seminiferous tubules and normal spermatogenic cells. **B** and **C** CP (200 mg/kg) treated rats; showing small atrophied seminiferous tubules with severe testicular degeneration, and loss of germ cells lining several seminiferous tubules (black arrow), spermatid giant cells formation (red arrow) and marked interstitial edema (blue arrow). **D** Treated with chrysin (25 mg/kg), showing congestion of interstitial blood vessel (black arrow). **E** Treated with chrysin (50 mg/kg), showing mild degeneration of sparse seminiferous tubules (black arrow). **F** treated with chrysin (100 mg/kg), showing obvious improvement with re-establishment of normal spermatogenic series and sperm production (scale bar, 100 μm)

All above results were confirmed with the histopathological examination. Microscopically, testes of normal control rats revealed the normal histoarchitecture of active seminiferous tubules and normal spermatogenic cells (Fig. 6A). In adverse, CP induced remarkable histopathological lesions characterized by small atrophied seminiferous tubules, severe testicular degeneration, and necrosis of spermatogonial cells lining seminiferous tubules with formation of multinucleate spermatid giant cells (Fig. 6B and C). Examined sections also exhibited complete loss of germ cells lining several seminiferous tubules, marked interstitial edema (Fig. 6C), and thickening of testicular basement membranes. Otherwise, testes of rats treated with low dose of chrysin (25 mg/kg) showed improvement in the induced histopathological lesions which summarized as mild testicular degeneration with incomplete spermatogenic series of some seminiferous tubules, few spermatozoa present in the lumen of some seminiferous tubules and congestion of interstitial blood vessels (Fig. 6D). Meanwhile, testicular tissues of rats from chrysin (50 mg/kg) treated group revealed mild degeneration of sparse seminiferous tubules (Fig. 6E). Furthermore, testes of rats treated with of chrysin (100 mg/kg) revealed obvious improvement with re-establishment of normal spermatogenic series and sperm production (Fig. 6F).

Johnson scoring of testicular tissues of different experimental groups is shown in Table 1. Briefly, there was a significant histopathologic decrease in Johnsen’s score of the testes of CP treated group as compared to control group. On the other hand, the groups treated with three doses of chrysin exhibited a significant decrease in the testicular lesion scoring in a dose-dependent manner, and the group treated with chrysin (100 mg/kg) showed normal score as control group.

**Table 1:** Effect of chrysin on testicular Johnson’s scoring of cyclophosphamide-induced testicular injury

Group	Normal control	CP (200 mg/kg)	CP + chrysin (25 mg/kg)	CP + chrysin (50 mg/kg)	CP + chrysin (100 mg/kg)
Johnson’s scoring	10.00±0.00	2.00±1.05*	7.10±0.79*	8.80±0.79^@^	9.80±0.42^@^

Data are presented as mean ± SD. *Significant difference from normal control; ^@^Significant difference from CP

## Discussion

This study investigated the potential for chrysin, a natural compound, to counteract the negative impacts of CP, a chemotherapy drug, on various testicular functional parameters in rats. Here, the impact of both treatments on serum hormone levels, testicular inflammatory markers, oxidative stress indicators, and sperm quality were extensively assessed. CP administration results in substantial testicular damage, as evidenced by a comprehensive array of functional, biochemical, and molecular markers. These results are in accordance with previous studies (Namasivayam et al. 2006; Hosseini et al. 2018; Matoso et al. 2019; Briseño-Bugarín et al. 2021; Ebokaiwe et al. 2022; van den Boogaard et al. 2022; Manal 2024).

CP treatment in the current study resulted in a decrease in the quality of the semen evidenced by a 29% reduction in sperm count and a 23% decrease in progressive motility. These effects are explained by dysregulated gonadotropin production, FSH and LH, as a result of decreased Leydig and Sertoli cell maturation, which lowers the mass of differentiated spermatogenic cells and reduces testosterone release (Ramaswamy and Weinbauer 2014).

Furthermore, this study explored the effects of CP on testosterone, inhibin B, and FSH levels. These hormones play crucial roles in male reproductive function (testosterone), regulating follicle development in females (inhibin B), and stimulating sperm production (FSH). Moreover, CP increased testicular cholesterol levels, the primary substrate from which all steroid hormones are synthesized, including testosterone, reaching 2.5-fold the normal levels. The process begins with the transport of cholesterol into the mitochondria of steroidogenic cells where it is converted into pregnenolone through a series of enzymatic reactions. Pregnenolone then undergoes further enzymatic modifications to eventually form testosterone (Miller and Bose 2011).

These findings are reinforced by the research of Ahmed et al. (2015) and highlighted in our investigation by histological findings that showed the breakdown of Leydig cells. Reduced spermatogenesis and the observed deterioration in sperm characteristics are caused by decreased testosterone levels, deteriorated spermatogonia, atrophied STs, and degenerated spermatocytes and spermatids (Sciorio et al. 2024). These alterations are consistent with CP-induced DNA damage, whereas CP breaks down lysosomal membranes by freeing their hydrolytic enzymes in the cytoplasm, which causes the target DNA material to lyse and dissolve visibly (Marinaro and Schlegel 2023).

CP toxicity was attributed to the activation of testicular oxidative stress, apoptotic pathways, and inflammatory cascades (Salama et al. 2020). Oxidative stress may be the cause of decreased sperm motility (Walczak-Jedrzejowska et al. 2013). In addition, this abnormal state of oxidative stress promotes mitochondrial permeability transition pore depolarization, which in turn activates the mitochondrial apoptotic pathway (Zhang et al. 2023). Interestingly, oxidative stress influences the regulation of apoptotic pathways by modulating the activity of BAX and BCL2, key proteins involved in cell death and survival. Elevated reactive oxygen species (ROS) levels enhance the expression and activation of BAX, promoting mitochondrial dysfunction and apoptosis, while concurrently downregulating BCL2, thus reducing its anti-apoptotic effects. The imbalance in the BAX/BCL2 ratio under oxidative stress conditions tilts the scale towards cell death, highlighting the critical role of oxidative stress in apoptosis regulation (Qian et al. 2022). Hereby, CP increases the expression of target genes Bax and decrease the Bcl2 this data suggests accelerated apoptosis (Salama et al. 2020).

CP treatment disrupted the body’s antioxidant balance, as evidenced by increased MDA levels and decreased GSH and GPx4 activity. This finding suggests that CP may induce oxidative stress, a cellular imbalance that can harm tissues, whereas the impact of CP on MDA, a marker of lipid peroxidation, as well as GSH levels and GPx4 enzyme activity are both vital components of the antioxidant system. It has been previously shown that CP dramatically elevated the testicular oxidative stress evidenced by increase in the MDA levels and decrease in the GSH levels (Abu-Risha et al. 2021). Oxidative stress wreaks havoc on testicular function through several mechanisms. ROS directly attack sperm DNA, causing mutations that hinder fertilization. Additionally, these free radicals damage the sperm membrane’s fatty acids, compromising its integrity and motility (Dutta et al. 2019). Furthermore, oxidative stress disrupts spermatogenesis by triggering programmed cell death in developing sperm cells, ultimately leading to a lower sperm count. It can also compromise the blood-testis barrier, allowing harmful substances to infiltrate and damage sperm cells (Aitken and Roman 2008; Luaces et al. 2023). It can also damage the Sertoli cells, crucial for sperm development, hindering their ability to nurture sperm maturation. These detrimental effects contribute to various testicular pathologies, including male infertility (Ayad et al. 2022).

On the other hand, chrysin demonstrates significant protective effects against CP-induced testicular injury. Interestingly, the investigated mechanistic lines of chrysin involved multi-channel pathways including ameliorating oxidative stress and inflammation and subsequent apoptosis and genotoxocity.

According to certain studies, chrysin therapy may mitigate oxidative stress by using its antioxidant properties (Li et al. 2022; Soliman et al. 2022; Ye et al. 2022; Saleh et al. 2023). Chrysin showed dose-dependent improvement in these parameters. At 100 mg/kg, chrysin increased testis weight by 18%, live percentage by 6%, decreased dead percentage by 12%, and increased fructose content by 16% close to control levels. Significant improvements were observed in sperm count, with a 22% increase, and non-progressive motility, with a 14% increase. However, progressive motility remained unaffected across all groups. This flavonoid effectively prevents the detrimental effects of CP on serum parameters such as testosterone, inhibin B, and FSH.

Chrysin administration boosted the Leydig cells’ synthesis of testosterone in both in vitro and in vivo experiments, which is consistent with our findings (Soliman and Aldhahrani 2022; Abadi et al. 2023). Remarkably, a recent assessment found that giving normal rats a 50 mg/kg dose of chrysin improved their sperm motility, concentration, and abnormal sperm rate (Ciftci et al. 2012; Soliman and Aldhahrani 2022). Chrysin emerged as a potential protector against CP’s disruptive effects on hormone levels. The study observed significant reductions in testosterone and inhibin B, along with a concerning increase in FSH, following CP treatment. Interestingly, chrysin administration displayed a dose-dependent response, effectively counteracting these negative changes. Higher chrysin doses led to progressively stronger positive effects, even restoring inhibin B levels to normal in the highest dose group. Chrysin treatment also reduced testicular cholesterol levels in a dose dependent manner.

Chrysin also shows that it may prevent the decline in reproductive hormones, as documented in this and previous research (Jana et al. 2008; Cormier et al. 2018). Chrysin has also been demonstrated to influence Leydig cell activity by raising the sensitivity of the cell to cAMP stimulation (Jana et al. 2008).

Fortunately, research on antioxidant supplementation and therapies to manage ROS or boost antioxidant defenses, viz., chrysin, holds promise in mitigating oxidative stress-induced testicular injury. Chrysin administration prevented these effects in a dose-dependent manner. It reduced MDA levels, a marker of damage, and increased both GSH levels and GPx4 activity, key players in antioxidant defense. The highest dose achieved a nearly 65% decrease in MDA, a 2.8-fold increase in GSH, and a more than 3-fold increase in GPx4 activity. These results suggest that chrysin may have potent antioxidant properties that could help protect against CP-induced oxidative stress. Previous studies have shown that chrysin protects against CP-induced toxicity in rats by modulating levels of MDA. Chrysin administration also increases the activities of antioxidant enzymes such as superoxide and catalase, as well as GSH levels (Ye et al. 2022).

Analogously, numerous investigations show that the injection of chrysin boosted the activity of antioxidant enzymes and shielded the tissue from oxidative stress (Pushpavalli et al. 2010; Singh et al. 2014). In this context, it is plausible to argue that the injection of chrysin may lower the levels of lipid peroxidation and shield the testis from oxidative stress if lipid peroxidation arises in the testes due to various causes, such as exposure to chemicals or ischemia. Furthermore, in rat testis tissue, our investigation demonstrated that chrysin had antioxidative and free-radical-scavenging properties (Kazaz et al. 2021). Chrysin inhibited oxidative stress in the D-gal-induced mouse model, and its effects may have been mediated through the inhibition of ROS (Li et al. 2022).

Exposure to CP was found to induce inflammation (Abu-Risha et al. 2021). While inflammation is a normal immune response, chemotherapeutic agents can trigger excessive or chronic inflammation, disrupting testicular function. When immune cells like macrophages swarm the testes, they release inflammatory mediators that damage sperm cells and hinder sperm production. This infiltration can also compromise the blood-testis barrier, a protective shield for developing sperm (Dutta and Sengupta 2021). This study examined the effects of CP on IL-6, a key inflammatory marker. The findings revealed a dramatic increase in IL-6 levels following CP treatment, suggesting a potential pro-inflammatory state.

Positively, managing underlying conditions give a chance for reducing inflammation and thus improving testicular health. Chrysin administration offered encouraging results. It reduced IL-6 levels in a dose-dependent manner, with the highest dose achieving a nearly 70% reduction. These findings suggest that chrysin may have anti-inflammatory properties that could be beneficial in mitigating CP’s inflammatory effects. According to previous research, chrysin therapy dramatically and concentration-dependently reduced the rise in TNF-α, IL-1β, and IL-6 levels in mice given D-gal, suggesting that chrysin alleviated strong expression of inflammatory markers in the ovaries (Li et al. 2022).

Flavones, including chrysin, are known for having highly promiscuous effects, which raises the possibility of these compounds being classified as pan-assay interfering substances (PAINS) (Baell and Walters 2014). This classification suggests that some effects might result from non-specific interactions rather than targeted actions. To address this, specific biochemical markers related to testicular injury and oxidative stress were carefully selected. While the PAINS classification is important, the protective effects of chrysin observed align with its known antioxidant and anti-inflammatory properties.

Additionally, CP showed a decrease in the anti-apoptotic marker BCL2 as well as an increase in the BAX levels (Abu-Risha et al. 2021). Conversely, chrysin reduces inflammatory marker, BAX while increasing the anti-apoptotic marker, BCL2. As previously demonstrated, chrysin prevents apoptosis by upregulating Bcl-2 mRNA expression and downregulating pro-apoptotic mRNA expression indicators (Talebi et al. 2021). According to Wolter et al. (1997) and Yang et al. (1997), the overexpression of Bcl2 shields the cells from toxic damage, prevents cell death, downregulates BAX, and prevents apoptosis. By upregulating Bcl-2 mRNA expression and downregulating BAX expression, chysin was able to exert its anti-apoptotic factor impact (Thangarajan et al. 2016; Talebi et al. 2021). Furthermore, following chrysin administration, the elevated Bax and downregulated Bcl-2 expression brought on by D-gal stimulation were attenuated, suggesting that chrysin decreased D-gal-induced POF via inducing cell death (Li et al. 2022).

CP treatment reduced the levels of TfR1 and IREB2 and liprin highlighting its detrimental impact on testicular function, particularly through the process of ferroptosis, leading to oxidative stress, lipid peroxidation, and cell death in testicular tissue. This ferroptotic cell death mechanism further exacerbates testicular dysfunction, resulting in reduced spermatogenesis, decreased sperm quality, and lowered testosterone production (Shi et al. 2022).

Reduced TfR1 levels, which is essential for iron uptake in cells suggest impaired iron metabolism, is crucial for the production of enzymes and proteins involved in spermatogenesis and testosterone synthesis. IREB2 plays a crucial role in preventing ferroptosis by regulating iron metabolism. Reduced IREB2 levels can lead to an imbalance in iron homeostasis, promoting ferroptotic cell death and exacerbating the effects of reduced TfR1 levels (Wang et al. 2020). Moreover, liprin is involved in cellular transport and signaling. Its decreased levels with CP indicate disruptions in cellular communication and structural integrity within the testes. Proper signaling is necessary for the coordination of spermatogenesis and the maintenance of Sertoli and Leydig cell functions. Disruption in these processes can lead to impaired sperm development and reduced testosterone production. Ferroptosis is characterized by the accumulation of lipid peroxides, which can be exacerbated by impaired signaling pathways and cellular transport mechanisms, leading to increased susceptibility to oxidative stress (Feng et al. 2023).

Chrysin enhances sperm count and quality, along with increasing testicular protein expression of TfR1, IREB2, and liprin in a dose-dependent manner. Chrysin treatment increased TfR1 protein expression. Similar to TfR1, chrysin increased IREB2 protein expression in a dose-dependent way. Unlike the other two proteins, chrysin at the lowest dose (25 mg/kg) had a negligible effect on liprin. However, higher doses increased liprin. By increasing the expression of these proteins, chrysin might influence iron metabolism or antioxidant pathways within the testes, thereby protecting testicular cells from ferroptosis-induced death. This, in turn, could contribute to improved sperm production and quality (Feng et al. 2023; Su et al. 2023). Overall, these findings highlight chrysin’s potential to promote testicular function by potentially regulating ferroptosis, a novel area of research in male fertility.

## Conclusion

**Fig. 7:**
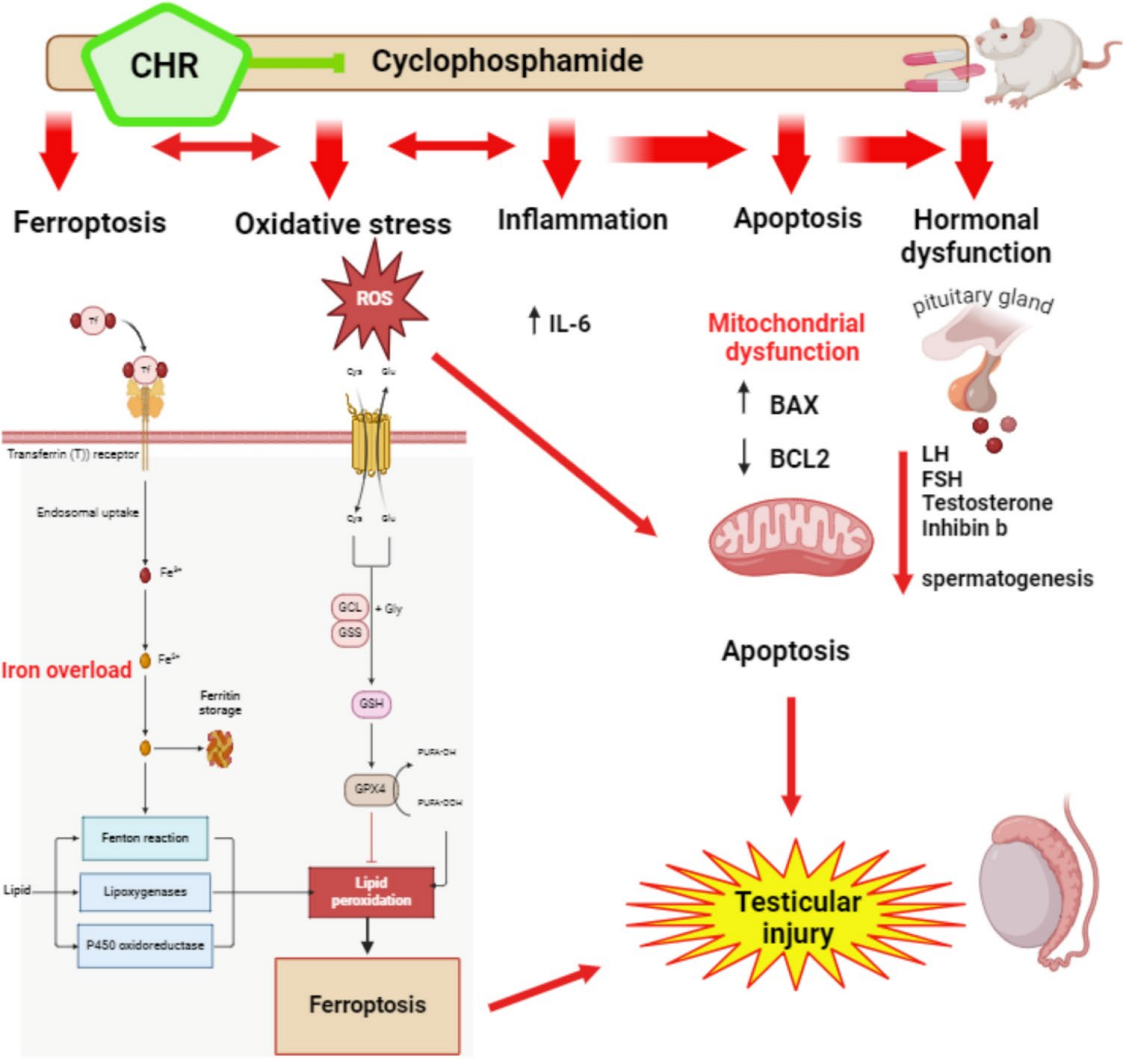
The proposed mechanism by which chrysin exhibits potent protective effects against CP-induced testicular injury. CP treatment caused severe damage to the testicular tissue, as evidenced by depleted levels of testosterone, inhibin B, glutathione, and GPx4. Additionally, CP treatment resulted in profound elevations in FSH, cholesterol, lipid peroxidation, inflammation, and apoptotic signaling, along with the suppression of iron regulatory proteins IREB2, TfR1, and liprin. Chrysin’s protective effects likely involve mitigating these detrimental changes, thereby preserving testicular function and integrity. Chrysin’s protective mechanisms likely include its antioxidant, anti-inflammatory, and anti-apoptotic activities, as well as its role in maintaining iron homeostasis

In conclusion, this study demonstrates that chrysin exhibits potent protective effects against CP-induced testicular injury in a rat model. CP treatment caused severe damage to the testicular tissue, indicated by depleted levels of testosterone, inhibin B, glutathione, GPx4 and profound elevations in FSH, cholesterol, lipid peroxidation, inflammation, apoptotic signaling and suppression of iron regulatory proteins IREB2, TfR1, and Liprin. These findings highlight chrysin’s novel spermatoprotective properties and ability to tackle CP’s complex multi-pronged gonadal toxicity. The mechanisms of chrysin likely involve its anti-oxidant, anti-inflammatory, and anti-apoptotic activities, along with maintaining iron homeostasis as illustrated in Fig. 7. Chrysin shows promise as an adjuvant to prevent chemotherapy-induced testicular injury and preserve male fertility. Further studies can optimize the dosage and explore the translational potential of these applications.

## Limitations of the study

Our study focused on chrysin’s protective effects against CP-induced testicular injury, but it lacked a direct comparison with mesna, a well-established agent for preventing CP-induced bladder and kidney toxicity. This omission limits the assessment of chrysin’s clinical relevance, and future research should explore the relative efficacy of these agents.

Additionally, chrysin, like other flavones, may be classified as a pan-assay interfering substance (PAINS) due to its potential for non-specific interactions. Although we selected specific biochemical markers to mitigate this concern, further research is needed to confirm chrysin’s specific effects and its potential as a therapeutic agent for CP-induced testicular injury.

## Data Availability

All source data for this work (or generated in this study) are available upon reasonable request.

## References

[CR1] Abadi ARR, Boukani LM, Shokoohi M, Vaezi N, Mahmoodi M, Gharekhani M, Kouchesfahani HM, Khaki AA (2023) The flavonoid chrysin protects against testicular apoptosis induced by torsion/detorsion in adult rats. Andrologia 2023:6500587

[CR2] Abu-Risha S, Mousa M, El Sisi AE (2021) Protective role of irbesartan against cyclophosphamide-induced testicular damage in rats via up-regulating PPAR-γ signaling and ameliorating NF-κB/NLRP3/IL-18 inflammatory axis. Life Sci 289:120218 34890588 10.1016/j.lfs.2021.120218

[CR3] Ahmed LA, El-Maraghy SA, Rizk SM (2015) Role of the KATP channel in the protective effect of nicorandil on cyclophosphamide-induced lung and testicular toxicity in rats. Sci Rep 5:14043 26403947 10.1038/srep14043 PMC4585895

[CR4] Aitken RJ, Roman SD (2008) Antioxidant systems and oxidative stress in the testes. Oxid Med Cell Longev 1:15–24 19794904 10.4161/oxim.1.1.6843 PMC2715191

[CR5] Ayad B, Omolaoye TS, Louw N, Ramsunder Y, Skosana BT, Oyeipo PI, Du Plessis SS (2022) Oxidative stress and male infertility: evidence from a research perspective. Front Reproduct Health 4:822257 10.3389/frph.2022.822257 PMC9580735 36303652

[CR6] Ayegbusi OS, Enye LA, Saka OS, Omoaghe AO (2023) Counteractive effects of extracts of Mangifera indica on testes of Wistar Rat exposed to cyclophosphamide. Heliyon 9:e19445 37674830 10.1016/j.heliyon.2023.e19445 PMC10477475

[CR7] Baell J, Walters MA (2014) Chemistry: chemical con artists foil drug discovery. Nature 513:481–483 25254460 10.1038/513481a

[CR8] Bancroft JD, Gamble M, Jones ML, Totty BA (2004) Theory and practice of histological techniques. Connective tissues and stains, 15thedn. Churchill Livingstone Publications, 139-200

[CR9] Briseño-Bugarín J, Hernández-Ochoa I, Araujo-Padilla X (2021) Phycobiliproteins ameliorate gonadal toxicity in male mice treated with cyclophosphamide. Nutrients 13:2616 34444776 10.3390/nu13082616 PMC8400975

[CR10] Busı MC, Yigitaslan S, Kaltus Z, Harmancı N, Eroglu E, Ozatık O, Kaya C (2023) The protective effect of taurine on cyclophosphamide-induced testicular toxicity in rats. Pak J Pharm Sci 36:1671–1676 38008966

[CR11] Ciftci O, Ozdemir I, Aydin M, Beytur A (2012) Beneficial effects of chrysin on the reproductive system of adult male rats. Andrologia 44:181–186 21486424 10.1111/j.1439-0272.2010.01127.x

[CR12] Cormier M, Ghouili F, Roumaud P, Bauer W, Touaibia M, Martin L (2018) Influences of flavones on cell viability and cAMP-dependent steroidogenic gene regulation in MA-10 Leydig cells. Cell Biol Toxicol 34:23–38 28455626 10.1007/s10565-017-9395-8

[CR13] Dutta S, Sengupta P (2021) Oxidative stress, testicular inflammatory pathways, and male reproduction. Int J Mol Sci 22:10043 34576205 10.3390/ijms221810043 PMC8471715

[CR14] Dutta S, Majzoub A, Agarwal A (2019) Oxidative stress and sperm function: a systematic review on evaluation and management. Arab J Urol 17:87–97 31285919 10.1080/2090598X.2019.1599624 PMC6600059

[CR15] Ebokaiwe AP, Obasi DO, Njoku RC, Osawe S (2022) Cyclophosphamide-induced testicular oxidative-inflammatory injury is accompanied by altered immunosuppressive indoleamine 2, 3-dioxygenase in Wister rats: Influence of dietary quercetin. Andrologia 54:e14341 34854117 10.1111/and.14341

[CR16] Farag OM, Abd-Elsalam RM, El Badawy SA (2021) Portulaca oleracea seeds’ extract alleviates acrylamide-induced testicular dysfunction by promoting oxidative status and steroidogenic pathway in rats. 21:122 10.1186/s12906-021-03286-2 PMC8045344 33853605

[CR17] Feng S, Tang D, Wang Y, Li X, Bao H, Tang C, Dong X, Li X, Yang Q, Yan Y, Yin Z, Shang T, Zheng K, Huang X, Wei Z, Wang K, Qi S (2023) The mechanism of ferroptosis and its related diseases. Mol Biomed 4:33 37840106 10.1186/s43556-023-00142-2 PMC10577123

[CR18] Hosseini A, Zare S, Borzouei Z, Ghaderi Pakdel F (2018) Cyclophosphamide-induced testicular toxicity ameliorate by American ginseng treatment: an experimental study. Int J Reprod Biomed 16:711–718 30775687 PMC6350852

[CR19] Jana K, Yin X, Schiffer RB, Chen JJ, Pandey AK, Stocco DM, Grammas P, Wang X (2008) Chrysin, a natural flavonoid enhances steroidogenesis and steroidogenic acute regulatory protein gene expression in mouse Leydig cells. J Endocrinol 197:315–323 18434361 10.1677/JOE-07-0282

[CR20] Kazaz İ, Demir S, Kerimoğlu G, Çolak F, Türkmen N, Akman A, Cekuc O, Mentese A (2021) Effect of chrysin on endoplasmic reticulum stress in a rat model of testicular torsion. J Investig Surge 35:1–6 10.1080/08941939.2021.2015489 34906035

[CR21] Khamis T, Hegazy AA, El-Fatah SSA, Abdelfattah ER, Abdelfattah MMM, Fericean LM, Arisha AH (2023) Hesperidin mitigates cyclophosphamide-induced testicular dysfunction via altering the hypothalamic pituitary gonadal axis and testicular steroidogenesis, inflammation, and apoptosis in male rats. Pharmaceuticals (Basel) 16:301 37259444 10.3390/ph16020301 PMC9966503

[CR22] Li X, Li X, Deng L (2022) Chrysin reduces inflammation and oxidative stress and improves ovarian function in D-gal-induced premature ovarian failure. Bioengineered 13:8291–8301 35311454 10.1080/21655979.2021.2005991 PMC9161991

[CR23] Ling H, Xiao H, Luo T, Lin H, Deng J (2023) Role of ferroptosis in regulating the epithelial–mesenchymal transition in pulmonary fibrosis. Biomedicines 11:163 36672671 10.3390/biomedicines11010163 PMC9856078

[CR24] Luaces JP, Toro-Urrego N, Otero-Losada M, Capani F (2023) What do we know about blood-testis barrier? current understanding of its structure and physiology. Front Cell Dev Biol 11:1114769 37397257 10.3389/fcell.2023.1114769 PMC10307970

[CR25] Manal AA (2024) Protective effect of apigenin against cyclophosphamide-induced testicular damage in mice by modulating inflammation, oxidative stress, and apoptosis and upregulating Nrf2/HO-1 pathway. J Biol Reg Homeostatic Agents 38:1079–1091

[CR26] Marinaro JA, Schlegel PN (2023) Sperm DNA damage and its relevance in fertility treatment: a review of recent literature and current practice guidelines. Int J Mol Sci 24:1446 36674957 10.3390/ijms24021446 PMC9860847

[CR27] Matoso V, Bargi de Souza P, Ivanski F, Romano MA, Romano R (2019) Acrylamide: a review about its toxic effects in the light of Developmental Origin of Health and Disease (DOHaD) concept. Food Chem 283:422–430 30722893 10.1016/j.foodchem.2019.01.054

[CR28] Miller WL, Bose HS (2011) Early steps in steroidogenesis: intracellular cholesterol trafficking. J Lipid Res 52:2111–2135 21976778 10.1194/jlr.R016675 PMC3283258

[CR29] Namasivayam E, Chiou T-J, Tzeng W-F, Chu S-T (2006) Cyclophosphamide treatment causes impairment of sperm and its fertilizing ability in mice. Toxicology 222:60–70 16517039 10.1016/j.tox.2006.01.027

[CR30] Parandin R, Ghowsi M, Dadbod A (2023) Protective effects of hydroalcoholic extract of Rosa canina L. fruit on cyclophosphamide-induced testicular toxicity in mice. Avicenna J Phytomed 13:7–17 36698735 10.22038/AJP.2022.20893 PMC9840777

[CR31] Pushpavalli G, Kalaiarasi P, Veeramani C, Pugalendi KV (2010) Effect of chrysin on hepatoprotective and antioxidant status in D-galactosamine-induced hepatitis in rats. Eur J Pharmacol 631:36–41 20056116 10.1016/j.ejphar.2009.12.031

[CR32] Qian S, Wei Z, Yang W, Huang J, Yang Y, Wang J (2022) The role of BCL-2 family proteins in regulating apoptosis and cancer therapy. Front Oncol 12:985363 36313628 10.3389/fonc.2022.985363 PMC9597512

[CR33] Ramaswamy S, Weinbauer GF (2014) Endocrine control of spermatogenesis: role of FSH and LH/ testosterone. Spermatogenesis 4:e996025 26413400 10.1080/21565562.2014.996025 PMC4581062

[CR34] Rezaei S, Hosseinimehr SJ, Zargari M, Karimpour Malekshah A, Mirzaei M, Talebpour Amiri F (2021) Protective effects of sinapic acid against cyclophosphamide-induced testicular toxicity via inhibiting oxidative stress, caspase-3 and NF-kB activity in BALB/c mice. Andrologia 53:e14196 34333791 10.1111/and.14196

[CR35] Salama RM, Abd Elwahab AH, Abd-Elgalil MM, Elmongy NF, Schaalan MF (2020) LCZ696 (sacubitril/valsartan) protects against cyclophosphamide-induced testicular toxicity in rats: role of neprilysin inhibition and lncRNA TUG1 in ameliorating apoptosis. Toxicology 437:152439 32197949 10.1016/j.tox.2020.152439

[CR36] Saleh DO, El-Nasr N, Fayez AM, Ahmed KA, Mohamed RA (2023) Uro-protective role of chrysin against cyclophosphamide-induced hemorrhagic cystitis in rats involving the turning-off NF-κB/P38-MAPK, NO/PARP-1 and STAT-3 signaling cascades. Chemico-biological Interact 382:110585 10.1016/j.cbi.2023.110585 37263553

[CR37] Sciorio R, Tramontano L, Adel M, Fleming S (2024) Decrease in sperm parameters in the 21st century: obesity, lifestyle, or environmental factors? An updated narrative review. J Personal Med 14:198 10.3390/jpm14020198 PMC10890002 38392631

[CR38] Shi H, Hou B, Li H, Zhou H, Du B (2022) Cyclophosphamide induces the ferroptosis of tumor cells through heme oxygenase-1. Front Pharmacol 13:839464 35264971 10.3389/fphar.2022.839464 PMC8899725

[CR39] Singh TG, Sharma A, Devi S (2014) Exploring the mechanisms of chrysin in combating Alzheimer’s disease: therapeutic perspectives. J Appl Pharm Sci 14(05):069–078

[CR40] Soliman MM, Aldhahrani A (2022) Chrysin abrogates gibberellic acid-induced testicular oxidative stress and dysfunction via the regulation of antioxidants and steroidogenesis- and apoptosis-associated genes. 46:e14165 10.1111/jfbc.14165 35383962

[CR41] Soliman MM, Aldhahrani A, Gaber A, Alsanie WF, Mohamed WA, Metwally MMM, Elbadawy M (2022) Ameliorative impacts of chrysin against gibberellic acid-induced liver and kidney damage through the regulation of antioxidants, oxidative stress, inflammatory cytokines, and apoptosis biomarkers. Toxicol Res 11:235–244 10.1093/toxres/tfac003 PMC8882807 35237428

[CR42] Su Y, Liu Z, Xie K, Ren Y, Li C, Chen W (2022) Ferroptosis: a novel type of cell death in male reproduction. Genes (Basel) 14:43 36672785 10.3390/genes14010043 PMC9858973

[CR43] Su Y, Liu Z, Xie K, Ren Y, Li C, Chen W (2023) Ferroptosis: a novel type of cell death in male reproduction. Genes 14:43 10.3390/genes14010043 PMC9858973 36672785

[CR44] Talebi M, Talebi M, Farkhondeh T, Simal-Gandara J, Kopustinskiene DM, Bernatoniene J, Samarghandian S (2021) Emerging cellular and molecular mechanisms underlying anticancer indications of chrysin. 21:214 10.1186/s12935-021-01906-y PMC8050922 33858433

[CR45] Thangarajan S, Ramachandran S, Krishnamurthy P (2016) Chrysin exerts neuroprotective effects against 3-nitropropionic acid induced behavioral despair—mitochondrial dysfunction and striatal apoptosis via upregulating Bcl-2 gene and downregulating Bax—bad genes in male Wistar rats. Biomed Pharmacother 84:627 10.1016/j.biopha.2016.09.070 27690136

[CR46] van den Boogaard WMC, Komninos DSJ, Vermeij WP (2022) Chemotherapy side-effects: not all DNA damage is equal. Cancers (Basel) 14:627 35158895 10.3390/cancers14030627 PMC8833520

[CR47] Walczak-Jedrzejowska R, Wolski JK, Slowikowska-Hilczer J (2013) The role of oxidative stress and antioxidants in male fertility. Centr Eur Urol 66:60–67 10.5173/ceju.2013.01.art19 PMC3921845 24578993

[CR48] Wang S, He X, Wu Q, Jiang L, Chen L, Yu Y, Zhang P, Huang X, Wang J, Ju Z, Min J, Wang F (2020) Transferrin receptor 1-mediated iron uptake plays an essential role in hematopoiesis. Haematologica 105:2071–2082 31601687 10.3324/haematol.2019.224899 PMC7395265

[CR49] Watcho P, Mpeck IR, Deeh Defo PB, Wankeu-Nya M, Ngadjui E, Bonsou Fozin GR, Kamtchouing P, Kamanyi A (2019) Cyclophosphamide-induced reproductive toxicity: beneficial effects of Helichrysum odoratissimum (Asteraceae) in male Wistar rats. J Integr Med 17:366–373 31420286 10.1016/j.joim.2019.07.002

[CR50] Wolter KG, Hsu YT, Smith CL, Nechushtan A, Xi XG, Youle RJ (1997) Movement of Bax from the cytosol to mitochondria during apoptosis. J Cell Biol 139:1281–1292 9382873 10.1083/jcb.139.5.1281 PMC2140220

[CR51] Yang J, Liu X, Bhalla K, Kim CN, Ibrado AM, Cai J, Peng TI, Jones DP, Wang X (1997) Prevention of apoptosis by Bcl-2: release of cytochrome c from mitochondria blocked. Science 275:1129–1132 9027314 10.1126/science.275.5303.1129

[CR52] Yang X, Chen Y, Song W, Huang T, Wang Y, Chen Z, Chen F, Liu Y, Wang X, Jiang Y, Zhang C (2022) Review of the role of ferroptosis in testicular function. Nutrients 14:5268 36558426 10.3390/nu14245268 PMC9785324

[CR53] Ye B, Ling W, Wang Y, Jaisi A, Olatunji OJ (2022) Protective effects of chrysin against cyclophosphamide-induced cardiotoxicity in rats: a biochemical and histopathological approach. Chem Biodivers 19:e202100886 35014174 10.1002/cbdv.202100886

[CR54] Yuan W, Sun Z, Ji G, Hu H (2023) Emerging roles of ferroptosis in male reproductive diseases. Cell Death Discov 9:358 37770442 10.1038/s41420-023-01665-x PMC10539319

[CR55] Zhang Z, Huang Q, Zhao D, Lian F, Li X, Qi W (2023) The impact of oxidative stress-induced mitochondrial dysfunction on diabetic microvascular complications. Front Endocrinol 14:1112363 10.3389/fendo.2023.1112363 PMC9941188 36824356

